# Sirolimus-Eluting Balloon for the Treatment of Coronary Lesions in Complex ACS Patients: The SELFIE Registry

**DOI:** 10.1155/2020/8865223

**Published:** 2020-10-16

**Authors:** Gianluca Caiazzo, Mario De Michele, Luca Golino, Vincenzo Manganiello, Luciano Fattore

**Affiliations:** ICCU, San Giuseppe Moscati Hospital, ASL CE, Aversa, Italy

## Abstract

**Background:**

Sirolimus-coated balloons (SCBs) represent a novel therapeutic option for both in-stent restenosis (ISR) and de novo coronary lesions treatment, especially in small vessels. Our registry sought to evaluate the procedural and clinical outcomes of such devices in a complex acute coronary syndrome (ACS) clinical setting.

**Methods and Results:**

We treated 74 consecutive patients with percutaneous coronary intervention (PCI) with at least 1 SCB used for ISR and/or de novo coronary lesion in small vessels at our institution. Sixty-two patients presented with ACS, and their data were included in our analysis. The mean age was 67 ± 10 years, and patients presenting with ST-elevated myocardial infarction (STEMI) were 14 (23%). De novo lesions were 52%, whereas ISR was 48%. Procedural success occurred in 100% of the cases. At the 11 ± 7 months follow-up, major adverse cardiovascular events (MACEs) were 3 (4.8%). Cardiovascular death (CD) occurred in 1 (1.6%) patient and myocardial infarction (MI) in 2 patients (3.2%) as well as ischemia-driven target lesion revascularization (TLR). One probable subacute thrombosis occurred (1.6%) with no major bleedings. In a subgroup analysis, the incidence of MACE did not show significant differences between patients treated for de novo lesions and ISR (HR: 0.239; CI 95%: 0.003–16.761, *p*=0.509).

**Conclusions:**

In the SELFIE prospective registry, SCB showed a good safety and efficacy profile for the treatment of coronary lesions, both ISR and/or de novo in small vessels, in a complex ACS population of patients at the 11 ± 7 months follow-up.

## 1. Introduction

Drug-eluting stents (DES) are the gold standard devices for percutaneous treatment of coronary artery stenoses. Second-generation DES have shown superior safety and effectiveness profiles when compared to bare-metal stents (BMS) and first-generation DES [1, 2]. However, late stent thrombosis and restenosis still represent an issue, with a hazard of nearly 2% per year [3]. For this reason, the “leave nothing behind” concept has rapidly grown in the field of coronary interventions in the last decade, making room for extensive use of bioresorbable scaffolds and drug-coated balloons (DCB) in the clinical scenario. Unlike bioresorbable scaffolds which have temporarily been abandoned after initial promising findings [4], DCBs represent a well-established therapeutic tool for the treatment of coronary artery stenoses, with the “leave nothing behind” as the first-line strategy (stent implanted only as a bailout for the treatment of suboptimal result after the DCB) [5]. In particular, remarkable results have been reported with DCBs use in the ISR setting, which made possible their introduction with a class I recommendation in the European Society of Cardiology Guidelines for Myocardial Revascularization [6]. DCBs have also shown some degree of effectiveness in de novo coronary lesions and particularly in the small vessels setting [7–9]. Nowadays, the vast majority of DCBs available on the market are paclitaxel-coated, since paclitaxel, with its lipophilic property, guarantees rapid cellular uptake with a homogeneous distribution, allowing for a lasting effect on smooth muscle cells. However, in 2016, a new sirolimus-coated balloon (SCB, Magic Touch®, Envision Scientific PVT, India) obtained the CE mark, and little clinical evidence on its effectiveness have been produced to date, mainly in stable settings [10].

## 2. Methods

The sirolimus-eluting balloon for complex ACS patients (SELFIE) registry is a prospective single-center registry including ACS patients with at least one lesion treated with the SCB between April 2018 and May 2020. The aim of our single-center prospective registry was to test the procedural and clinical behavior of the SCB in a real-world scenario of acute coronary syndrome (ACS). The present study was conducted in accordance with the Declaration of Helsinki. All patients provided informed consent for both the procedure and subsequent data collection and analysis.

### 2.1. Device

The sirolimus-coated magic touch balloon is a new generation rapid-exchange monorail balloon with a distal tip of 0.016″ and a rigid hypotube. Sirolimus has a wider therapeutic window than paclitaxel; in this device, it is incapsulated in a protective phospholipid package of nano-sized drug particles of 100–300 nm diameter, which allows the diffusion and penetration of the drug into the arterial wall during balloon inflation, overcoming its low lipophilicity (Nanolutè® technology) [11]. The drug-carrier unit is uniformly distributed on the balloon surface through the use of a spray coating. Available balloon sizes range between 1.5 and 4.0 mm in diameter and 10–40 mm in length.

### 2.2. Study Population

**Figure 1:**
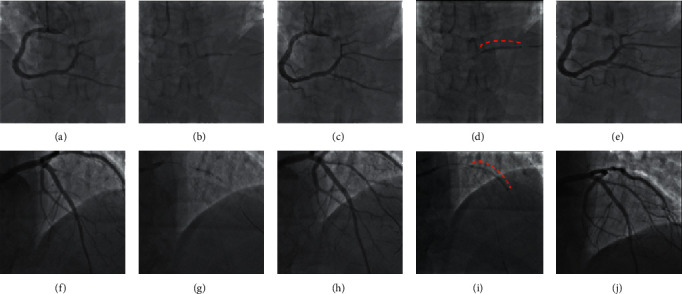
STEMI patients undergoing PCI with the SCB of de novo lesions in small vessels. Upper panels ((a)–(e)): total thrombotic occlusion of proximal posterolateral branch of the RCA (a) in a patient with inferior STEMI treated with a 2.25 × 12 mm balloon ((b) and (c)) and 2.25 × 30 mm SCB (d) with good TIMI 3 final result (e). Lower panels ((f)–(j)): total thrombotic occlusion of proximal first diagonal branch (f) in a patient with lateral STEMI treated with a 2.25 × 15 mm balloon ((g) and (h)) and 2.25 × 30 mm SCB (i) with good TIMI 3 final result (j).

**Figure 2:**
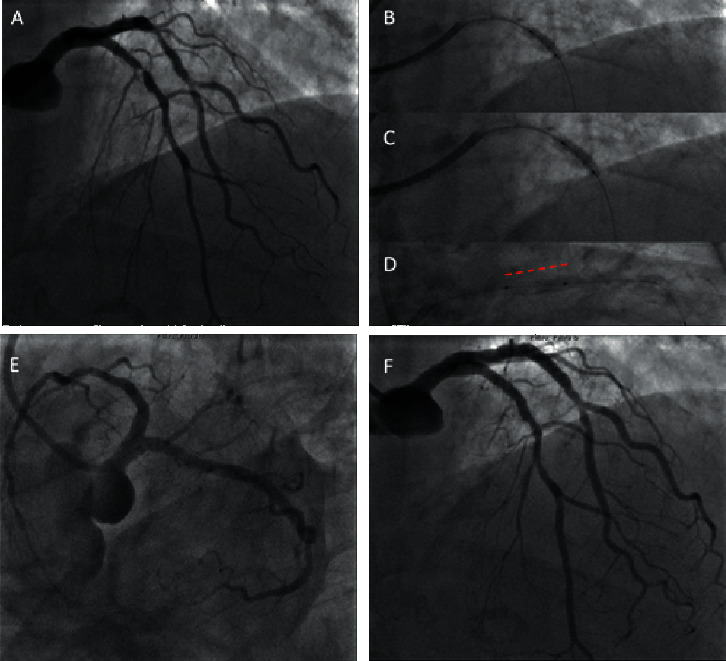
STEMI patient undergoing PCI with the SCB of ISR lesion on LAD. A patient with anterior STEMI for ISR on LAD (a) underwent PCI with a 2.75 × 12 mm noncompliant balloon (b), 3.0 × 15 mm noncompliant balloon (c), and 3.0 × 15 mm SCB (d), with good final angiographic result and TIMI 3 flow ((e) and (f)).

The SELFIE registry included the whole spectrum of ACS clinical presentations and a wide range of lesion types (Figures 1 and 2). Indeed, in-stent restenosis, small vessels, thrombotic lesions, long lesions, calcific stenosis, and bifurcation lesions were treated. Exclusion criteria were

- Vessel dimensions exceeding those of the device tested and/or balloon sizes not available at the moment of the procedure
- De novo lesions located within the left main coronary artery and/or proximal epicardial main vessels
- Patients who did not give written consent to be included in the registry

For every study participant, demographic, clinical, and procedural data were prospectively collected into a dedicated database, which included follow-up data. Clinical follow-up was achieved for all subjects by clinic visit or telephone interview.

### 2.3. Study Procedure

**Figure 3:**
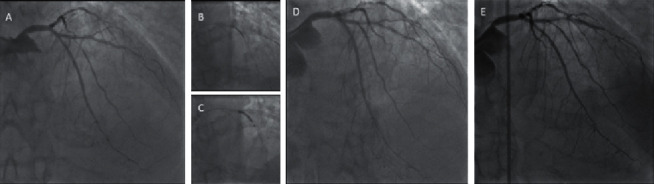
NSTEMI patient undergoing OCT-guided PCI with the SCB of ostial ISR on RCA. A patient with NSTEMI showing significant stenoses of the distal RCA with significant ISR at the ostium (a). All lesions were predilated with a 2.0 × 20 noncompliant balloon ((b) and (c)). A linear dissection became evident in the distal segment (d) and was covered with a 2.25 × 35 mm DES (e) postdilated with a noncompliant 2.5 × 12 mm balloon (f). The OCT pullback of the ostial ISR revealed a typical fibrotic pattern of neointimal hyperplasia causing significant restenosis (g). The lesion was treated with several inflations of a 3.5 × 15 mm noncompliant balloon at high atmospheres (h) and with a 3.5 × 15 mm SCB afterward (i). Final OCT pullback (j) showed a significant improvement of the MSA with a small neointimal dissection (see asterisk).

**Figure 4:**
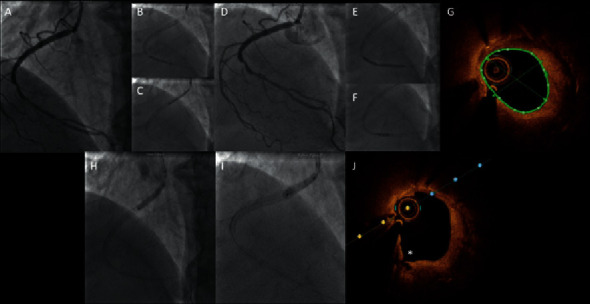
NSTEMI patient undergoing PCI with the SCB of de novo lesion with a 3-month angiographic follow-up. A patient with NSTEMI and a significant stenosis in the proximal segment of a collateral branch (small diameter but good extension) of the I diagonal (a); the lesion was treated with a 2.0 × 12 mm noncompliant balloon (b) and a 2.25 × 15 mm SCB (c) with good final angiographic result and TIMI 3 flow (d). The angiographic follow-up at 3 months is reported (e) with no restenosis nor thrombosis of the treated lesion.

The procedure was performed according to international guidelines. Vessel size and lesion length were assessed by both visual estimation and quantitative coronary angiography (QCA) analysis; discrepancies were discussed and resolved among operators. Careful lesion preparation was performed by using semicompliant or noncompliant balloons, and a 1 : 1 balloon-to-artery ratio was recommended. Inflation time for the SCB was 60 seconds at 6–8 atmospheres, and a second inflation was allowed at operator discretion. During the procedure, intravenous heparin (70–100 units/kg) was administered after sheath insertion to maintain an activated clotting time >250 seconds. Dual antiplatelet therapy with aspirin 100 mg once a day and ticagrelor 90 mg twice a day was recommended for 12 months following guidelines indications and at least for 1 month in case of need of suspension [12]. GP IIb/IIIa inhibitors were used at operators' discretion in case of plaques with high thrombus burden. Intracoronary imaging use was also left to operators' discretion (Figure 3). For each treated lesion, final angiographic result was considered satisfactory when the residual stenosis did not exceed 50%. Angiographic follow-up was not mandatory, unless clinically indicated (Figure 4).

### 2.4. Study Endpoints

The primary study endpoint was procedural success defined as final diameter stenosis <50% with 3 TIMI flow. Secondary endpoints were major adverse cardiovascular events (MACEs) at the longest follow-up available. MACEs were defined as a composite of cardiovascular death, myocardial infarction (MI), and target lesion revascularization (TLR), the single determinants of MACE at the longest follow-up. MI was defined according to the fourth universal definition [13]. TLR was defined as repeat PCI or coronary artery bypass grafting for the target segment or in the adjacent proximal or distal 5 mm segments. The impact of the type of lesion treated with DCB (ISR, de novo lesions) on the outcome was also evaluated.

### 2.5. Statistical Methods

Continuous variables were expressed as mean values ± SD, and values were reported as numbers with relative percentages of standard deviation. *p* values less than 0.05 were considered statistically significant. Cumulative event rates were analyzed using the Kaplan–Meier method, and the rate differences among the groups were estimated using the log-rank test. Cox regression analysis was performed to determine risk factors for MACE during the follow-up. All statistical analyses were performed using SPSS version 25.0 (IBM Corp. Armonk NY, USA).

## 3. Results

**Figure 5:**
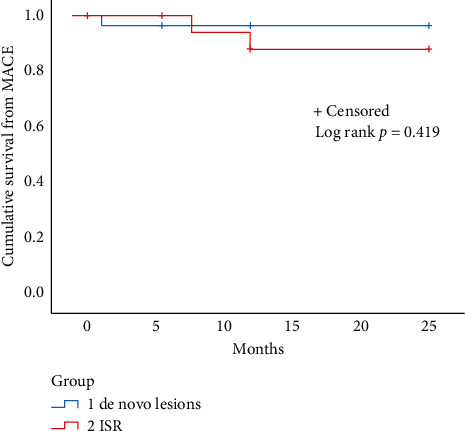
Kaplan–Meier curve of survival from the secondary study endpoint, MACE, at the longest available follow-up. MACE = major cardiovascular events.

Between April 2018 and May 2020, a total of 74 patients underwent PCI with use of a sirolimus-coated balloon. Patients presenting with ACS were 62 (84%); among these, 47 (76%) were males, 25 (40%) were diabetics, and the clinical presentation was STEMI in 23% of cases. Population characteristics are reported in Table 1. Seventeen patients (27.4%) received GP IIb/IIIa inhibitors during the procedure, and ISR and de novo lesions were, respectively, 48% and 52% of lesions types. Lesion characteristics and procedural aspects are shown in Table 2. Mean DCB length was 18 ± 5 mm, and the diameter was 2.6 ± 0.6 mm. Balloon-induced dissections of the target lesion occurred in 8 cases (13%) and were all type A or B (NHLBI classification); however, in 4 cases (6.4%), a stent covering the dissected segment was deployed. No intraprocedural complications or adverse events were observed. Procedural success, the primary endpoint, was reached in 100% of patients. The mean follow-up was 11 ± 7 months, and the procedural success, primary study endpoint, was achieved in 100% of the lesions. The secondary endpoint MACE occurred in 3 patients (4.8%). Two patients experienced MI and subsequent TLR, while one cardiovascular death occurred. No cases of abrupt vessel/thrombotic closure at lesion site were recorded. Clinical outcomes at follow-up are shown in Table 3. The cardiovascular death event occurred in one patient, which experienced acute heart failure due to severe aortic valvular stenosis (the patient was waiting for a scheduled TAVI procedure). Two MIs with subsequent re-PCI occurred in two patients treated with the SCB for ISR; no intracoronary imaging was performed, and it has not been possible to exclude stent underexpansion. In one of these patients, the re-MI was represented by an inferior STEMI with acute thrombosis of a previously implanted stent in the mid-RCA treated two months before with the SCB for ISR. We undertook a subanalysis of the data comparing patients treated for ISR and patients treated for de novo lesions and did not observe a significant difference in the MACE rate (HR: 0.239; CI 95%: 0.003–16.761, *p*=0.509) (Figure 5).

## 4. Discussion

The SELFIE registry is the first observational prospective study with SCBs in a population of ACS patients, confirming the safety and effectiveness of these devices in such a complex clinical setting. In our study, MACEs were 4.8%, which are even lower than those reported in previous studies with both sirolimus- and paclitaxel-coated balloons used in lower-risk populations of patients. Lower TLR and MI rates were also found, which is reassuring, accounting for the higher incidence of adverse events related to the ACS setting. Of note, at subgroups analysis, the incidence of MACE was similar between patients with de novo lesion and ISR. Drug-coated balloons represent an established tool in the panorama of vascular interventions, and the “leave nothing behind strategy” (DCB angioplasty with stent implantation only as a bailout for suboptimal results) represents the most appealing and reliable technique in this setting [5]. Paclitaxel-coated balloons have widely demonstrated their safety and efficacy profiles in terms of both procedural and clinical outcomes in different clinical settings [9, 14]. Among lesion settings, ISR and small vessels represent the most interesting fields of application for DCBs because of the attractive possibility of sparing adjunctive metal, respecting vessel anatomy, and reducing the intraluminal bulk. In line with this, we decided to limit the use of DCB to ISR and de novo lesions located in small vessels, avoiding epicardial proximal de novo lesions treatment. As a matter of fact, the majority of studies with DCBs have been conducted in small vessels. The BASKET-SMALL II trial [8] represents the largest study on small-vessel coronary artery disease, comparing a paclitaxel-coated balloon (SeQuent Please DCB, Braun Melsungen AG, Berlin, Germany) with second-generation everolimus- or paclitaxel-eluting stents (respectively, Xience stent, Abbott Vascular, Santa Clara, CA, USA, or the Taxus Element stent, Boston Scientific, Natick, MA, USA). The main finding was that DCB was noninferior to DES (MACE 8% vs. 9%) at the 12-month follow-up. The relatively recent introduction of SCBs replied to the need of testing a drug (the -limus family), which have reported better overall outcomes in the setting of drug-eluting stents when compared to paclitaxel [15]. Several small-sized studies have been conducted on the topic, mostly including a heterogeneous spectrum of clinical conditions and lesion settings. Since SCBs introduction in Europe in 2016, Cortese et al. first reported the procedural effectiveness of SCBs in 32 patients with coronary artery disease and a stable clinical presentation in 66% of cases [10]. Of note, 47% of patients underwent PCI for ISR, and procedural success was achieved in all patients. El-Mokdad et al. have shown the longest follow-up of patients treated with SCBs to date [16]. They included 408 patients, of which 45% had diabetes mellitus, and in 47% of cases, ACS was the indication to coronary angiography, with unstable angina accounting for 64.4% of all ACS. In this study, MACEs at 2 years were driven by TLR, which occurred in 3.2% of patients, with a trend toward better performances shown for de novo lesions when compared to the ISR setting (2.7% vs. 4.4%, respectively). Of note, our registry confirms such positive findings in an even more complex clinical scenario of ACS patients where STEMI and NSTEMI together represent 84% of the entire population of patients. To date, two studies have reported DCB to be noninferior to DES in the STEMI setting [14, 17]. Although there is still a lack of compelling evidence, young STEMI patients (23% in our study) represent an interesting setting for DCB use. It is not uncommon, indeed that these patients show noncalcified culprit lesions, which turn out to be ideal for simple ballooning and subsequent DCB with satisfactory final angiographic results. This is especially true for long lesions in small vessels where implanting a metallic stent might not be the best option for long-term outcomes. In line with this, is also reassuring that in our registry, no thrombotic complications occurred during the hospital stay and only one during the follow-up period not related to a de novo lesion. However, the small sample size of our registry suggests caution when commenting on these findings. Of note, the effect of the SCB on hard clinical endpoints will be assessed in the ongoing investigator-driven EASTBOURNE registry, which is actually enrolling patients until Q2 2020 and whose 12-month interim analysis is already available (accepted for publication in the Journal of Cardiovascular Medicine).

### 4.1. Limitations

All the limitations of a registry are to be considered here. As anticipated, the small population of patients included in our study does not allow any definitive conclusion from a clinical standpoint. Events adjudication was performed by single cardiologists participating at the study, but there was a common, prespecified, and per-protocol definition of all the events. In our registry, QCA measurement was obtained before PCI and at the very end of the procedure not allowing any meaningful insight regarding elastic recoil.

## 5. Conclusion

The SELFIE registry has been the first study evaluating the SCB for the treatment of coronary artery lesions in the high complexity setting of ACS patients. It provided reassuring findings on both procedural and clinical 12-month outcomes, similar to previous studies using the same device in different heterogeneous settings or using paclitaxel-coated balloons. Large-scale studies are needed to clarify the clinical relevance of such preliminary findings.

## Figures and Tables

**Table 1:** Patients characteristics.

Patients characteristics	*n* = 62

Age (mean ± SD)	67 ± 10
Male, *n* (%)	47 (76)
Hypertension, *n* (%)	47 (76)
Hypercholesterolemia, *n* (%)	36 (58)
Smoke, *n* (%)	21 (34)
Diabetes, *n* (%)	25 (40)
Family history, *n* (%)	14 (23)
Previous MI, *n* (%)	38 (61)
Previous PCI, *n* (%)	38 (61)
Previous CABG, *n* (%)	7 (11)
CKD, *n* (%)	11 (18)
LVEF (%) (mean ± SD)	48 ± 9
Clinical presentation, *n* (%)	
ACS	62 (100)
STEMI	14 (23)
NSTEMI	36 (58)
UA	8 (13)

SD, standard deviation; MI, myocardial infarction; PCI, percutaneous coronary intervention; CABG, coronary artery bypass grafting; CKD, chronic kidney disease; LVEF, left ventricular ejection fraction; ACS, acute coronary syndrome; STEMI, ST-elevated myocardial infarction; NSTEMI, non-ST-elevated myocardial infarction; UA, unstable angina.

**Table 2:** Procedural characteristics.

Procedural characteristics	*n* = 62

Small vessels (≤2.5 mm), *n* (%)	43 (69)
De novo lesions, *n* (%)	32 (52)
In-stent restenosis, *n* (%)	30 (48)
Lesion length (mm) (mean ± SD)	15 ± 4
Predilation, *n* (%)	61 (99)
DCB diameter (mm) (mean ± SD)	2.6 ± 0.6
DCB length (mm) (mean ± SD)	18 ± 5
DCB inflation time (sec) (mean ± SD)	62 ± 7
DCB inflation pressure (atm) (mean ± SD)	7 ± 2
Angiographic success, *n* (%)	62 (100)

SD, standard deviation; DCB, drug-coated balloon; atm, atmospheres.

**Table 3:** Incidence of clinical endpoints at 11 ± 7 months follow-up in the SELFIE registry.

Incidence of clinical endpoints at the 11 ± 7 months follow-up in the SELFIE registry	*n* = 62

MACE, *n* (%)	3 (4.8)
TLR, *n* (%)	2 (3.2)
MI, *n* (%)	2 (3.2)
CV death, *n* (%)	1 (1.6)
Acute thrombosis, *n* (%)	1 (1.6)
Bleeding, *n* (%)	0

MACE, major cardiovascular events; TLR, target lesion revascularization; MI, myocardial infarction; CV death, cardiovascular death.

## Data Availability

The data used to support this study are included within this article.

## Supplementary Materials

Supplementary Materials Supplementary file: flow chart of patient inclusion.
