# Supplementary figures and images for: Sirolimus-Eluting Balloon for the Treatment of Coronary Lesions in Complex ACS Patients: The SELFIE Registry
Source: J Interv Cardiol. 2020 Oct 16;2020:8865223. doi: 10.1155/2020/8865223 (PMC7586180; doi:10.1155/2020/8865223)

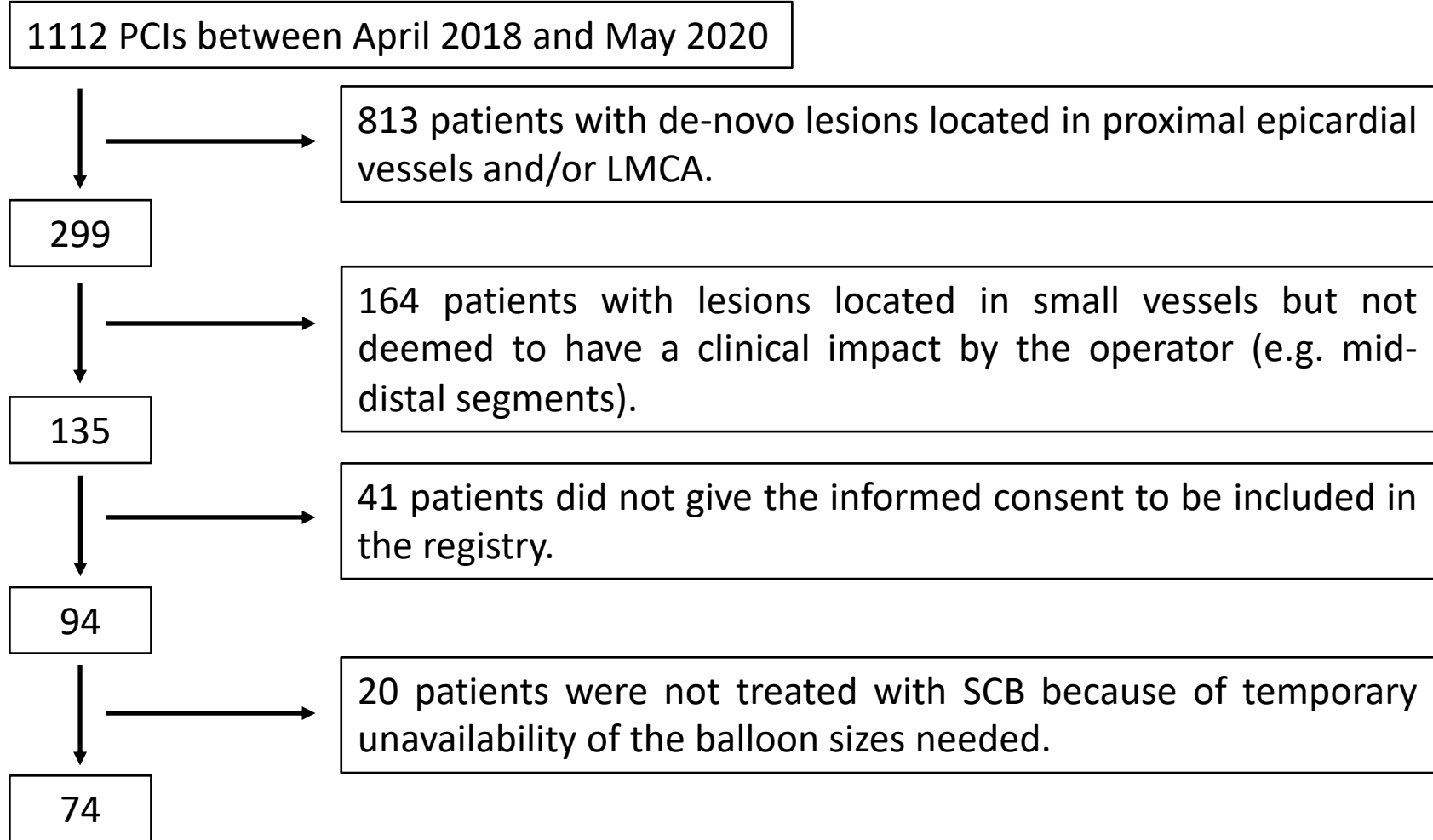

Supplement: Supplementary Materials — Supplementary file: flow chart of patient inclusion. [file 8865223.f1.pdf]
